# Aligning Natural Resource Conservation and Flood Hazard Mitigation in California

**DOI:** 10.1371/journal.pone.0132651

**Published:** 2015-07-22

**Authors:** Juliano Calil, Michael W. Beck, Mary Gleason, Matthew Merrifield, Kirk Klausmeyer, Sarah Newkirk

**Affiliations:** 1 Department of Ocean Sciences, University of California Santa Cruz, Santa Cruz, California, United States of America; 2 Global Marine Team, The Nature Conservancy, Santa Cruz, California, United States of America; 3 California Chapter, The Nature Conservancy, San Francisco, California, United States of America; University California Los Angeles, UNITED STATES

## Abstract

Flooding is the most common and damaging of all natural disasters in the United States, and was a factor in almost all declared disasters in U.S. history. Direct flood losses in the U.S. in 2011 totaled $8.41 billion and flood damage has also been on the rise globally over the past century. The National Flood Insurance Program paid out more than $38 billion in claims since its inception in 1968, more than a third of which has gone to the one percent of policies that experienced multiple losses and are classified as “repetitive loss.” During the same period, the loss of coastal wetlands and other natural habitat has continued, and funds for conservation and restoration of these habitats are very limited. This study demonstrates that flood losses could be mitigated through action that meets both flood risk reduction and conservation objectives. We found that there are at least 11,243km^2^ of land in coastal California, which is both flood-prone and has natural resource conservation value, and where a property/structure buyout and habitat restoration project could meet multiple objectives. For example, our results show that in Sonoma County, the extent of land that meets these criteria is 564km^2^. Further, we explore flood mitigation grant programs that can be a significant source of funds to such projects. We demonstrate that government funded buyouts followed by restoration of targeted lands can support social, environmental, and economic objectives: reduction of flood exposure, restoration of natural resources, and efficient use of limited governmental funds.

## Introduction

Flooding is the most common and damaging of all natural disasters in the United States [1]. Historically, floods have caused more economic loss to the nation than any other natural hazard and flooding has been a factor in almost all declared disasters in the U.S. [2]. Recently, several perilous and costly flood events including super storm Sandy and Hurricanes Irene, Ike and Katrina, have once again raised public awareness of the threats posed by coastal and riverine floods nationally. The average annual value of insured losses related to storms from 2007 to 2011 is $12.1 billion [3]. Direct flood losses in the U.S. in 2011 totaled $8.41 billion [4]. It is likely that with climate change the frequency of heavy precipitation will increase in some areas over the 21^st^ century, and that the return interval of floods will be shorter thus increasing the frequency of such events [5].

In 1968, the National Flood Insurance Program (NFIP) was created in response to wide spread demand for private insurance resulting from a series of catastrophic flood losses early in the twentieth century [6]. From its inception in 1968 until December of 2011, NFIP insured a total of 5.58 million policies and paid more than $38 billion in claims [7]. In addition to covering flood losses, one of the objectives of the NFIP was to encourage communities to adopt risk-minimizing measures by promoting floodplain management regulations to ultimately lower their flood risks [8], but for the most part, this has not occurred [9]. Partially because the NFIP has no strong provisions to guide development away from floodplains, many flood-prone areas of the United States are still subject to development [10].

As a consequence of existing and ongoing risky development, 1% of all NFIP policies are classified as Repetitive Loss Properties (RLPs) [7]–a detailed definition of RLPs and recent NFIP regulatory updates can be found in S1 Appendix [11]. According to the Federal Emergency Management Agency (FEMA), from 1978 to 2011, 166,368 Repetitive Loss Properties across the U.S. filed 496,178 claims resulting in more than $12.1 billion in payments, an average of $24,386 per claim [7]. One out of every ten Repetitive Loss Properties has received more money in reimbursements than the estimated market value of their property [1]. This startling fact suggests that purchasing RLPs for restoration to open space would save FEMA, and U.S. taxpayers, money.

Currently, FEMA administers three Hazard Mitigation Assistance (HMA) grant programs: (i) the Hazard Mitigation Grant Program (HMGP), focused on post-disaster reconstruction efforts including mitigation measures to reduce future risk; (ii) the Pre-Disaster Mitigation (PDM) grant program, supporting activities that reduce overall risk of future natural hazard events; and (iii), the Flood Assistance Mitigation (FMA) grant program, focused on reducing the number of NFIP claims [12]. In 2013, FEMA announced national grant opportunities for PDM and FMA of $23.7 million and $120 million respectively [13][14]. These funds may be used to acquire and remove or relocate structures away from risky areas [12], but to date that has not yet been a widespread activity [15]. Property acquisition and structure removal or relocation are the most permanent forms of mitigation, and are eligible activities under all three programs [12]. Nevertheless, from 1989 through 2011, only 28 acquisition projects were funded in California [15].

Protecting communities and private property from flooding has traditionally been accomplished through the use of fortifying structures (e.g., seawalls, dikes, and levees). However, natural habitats and ecosystems offer significant, and often overlooked and undervalued, protection, mitigating or buffering flood hazards [16–20]. Restored areas within the floodplain usually regain their natural function of attenuating floods and reduce cyclical flood damages [21]. The value of wetlands in protecting coastal communities against floods globally has been estimated at $6,923 per hectare per year [22].

While FEMA aims to protect people and properties from future floods and other disasters, the conservation community attempts to protect valuable threatened floodplain habitats and species. From the 1780’s to 1980’s California has seen the highest percent loss of coastal wetlands in the U.S., 91% [23]. This represents more than 4.5 million acres of wetlands lost [23]. Floodplain habitats and species are continuously under tremendous pressure from human impacts including: urban development, agricultural expansion, water quality issues, and habitat fragmentation from dams and other physical barriers [24].

Until very recently, hazard mitigation plans and conservation project plans did not explicitly recognize the flood protective value of natural habitats, even though this value has been well documented [19,20,25]. Increasingly, conservationists and risk managers are looking for approaches to accomplish multiple objectives with a single project [21,26,27]. In addition, conservation groups, in particular the National Wildlife Federation and The Nature Conservancy (TNC), have engaged in FEMA policy under the premise that flood risk response has direct impacts on natural resources [28,29]. However, to date, the development of tools for flexible, multi-objective flood exposure reduction and conservation prioritization permitting the identification of projects with the greatest likelihood of success, has been limited.

Recent studies have presented spatially explicit models of flood risk with diverse focuses. Some studies produced maps of flood prone areas based on terrain and hydraulic models [30,31], while others evaluated the costs and benefits associated with the use of land conservation as a flood mitigation strategy (including the impacts of flooding on housing values) [26,32,33]. However, to our knowledge, the study presented herein is the first detailed spatial analysis in the context of existing policy instruments which may be applied to achieve multiple benefits.

Here, we examine the potential to identify projects with multiple objectives in which flood exposure is reduced and conservation benefits are achieved. We test an approach for identifying developed and federally-insured lands that are prone to flooding and therefore not ideal for development, and where valuable natural resources, such as salmon habitat or estuaries, are also present. We examine whether, by defining appropriate flood exposure and conservation proxies, decision makers could identify and prioritize parcels and neighborhoods where flood exposure reduction and conservation objectives could be achieved simultaneously. Further, we describe federal funding programs that could be applied to achieve both flood mitigation and conservation objectives.

This study used coastal California as a model to evaluate the alignment between coastal flood mitigation and natural resource conservation because of the high number of RLPs and flood claims [7], and the many highly threatened floodplain habitats (e.g. saltmarsh) [23], including areas that support multiple salmonid species listed as either threatened or endangered [34]. By the end of 2011, more than 3,200 RLP owners in the state of California filed more than 9,000 claims against NFIP totaling $155.3 million [1]. The average claim payment was $21,200. At the same time, California hosts floodplain natural resources that provide important functions including water filtration, erosion control, pollution prevention and control, fish production, and recreation amongst many others [35]. Finally, California’s Multi-Hazard Mitigation Plan (SHMP) contains several explicit objectives to integrate hazard mitigation and environmental protection, calling for solutions that enhance natural processes with minimal negative impacts on natural ecosystems [36].

Additionally, we present a case study focused on the Sonoma County, where flooding has been a historical problem dating back to 1862 [37]. During the last twenty years, Federal and state disaster declarations were made following 8 significant flood events affecting the county [37]. Sonoma County, which occupies roughly 1% of the total area of California, accounts for 27.5% of all RLPs and 32% of all NFIP claims in the state. Sonoma County also represents an area of significant critical habitat for biodiversity conservation [38], thus representing a high priority area in coastal California for multi-benefit restoration and hazard mitigation projects.

## Materials and Methods

We used a weighted overlay spatial model developed in a desktop geographic information system (ESRI ArcGIS version 10.2) and applied it to the 21 coastal counties in California (total study area covers 94,500km^2^). Based on multiple flood exposure and conservation components (details below), we developed two indices: the Flood Exposure Index (FEI) and the Conservation Priority Index (CPI). The model calculates the spatial extent of overlap (km^2^) between selected indicators of conservation priorities and flood exposure. Each indicator of Conservation Priority and Flood Exposure received a score of 1 or 0, based on the occurrence or absence of the indicator as described in detail below. The FEI and the CPI have equal weight and vary from 0 to 10. These indices are intended to be qualitative and relative, rather than quantitative measures of any specific feature.

Using data from the FEMA’s Repetitive Flood Claims program and Digital Flood Insurance Rate Maps (DFIRM) [39], sea level rise projections from the California Climate Change Center [40], and spatial data on natural habitats and other indicators of conservation value in California, we examine the potential for projects with multiple management benefits in which flood exposure is reduced and conservation benefits are achieved.

### 1. Flood Exposure Index (FEI)

The FEI scores each grid cell in the study area according to multiple indicators of exposure to flooding events (grid cells are 50m by 50m or 0.0025Km^2^).

The FEI was calculated based on the following components:

- Whether or not the area is in either the 100-year or 500-year floodplain, based on FEMA’s digital Flood Insurance Rate Maps (DFIRM) [39]. DFIRMs are developed by FEMA based on detailed Flood Insurance Studies that include hydraulic, hydrologic and wave height analyses to determine the water surface elevations for the 100-year and 500-year floodplains [41,42].
- Whether or not the area is in California’s Coastal Zone, based on data from the National Oceanic and Atmospheric Administration (NOAA) [43].
- Sea Level Rise (SLR) projections at the year 2100, based on the “California Climate Change Scenarios Assessment” of 2009 [40,44,45]. The SLR components include areas projected to be below the mean high high water mark (MHHWM), and areas projected to be inside the 100-year floodplain at the year 2100 (based on a projected SLR of 1.4m). SLR projections are based on simulations of six global climate models which were forced by the greenhouse gases emissions scenario A2 (high emissions scenario) developed by the Intergovernmental Panel on Climate Change (IPCC) [46], and have a considerable level of uncertainty attached to them (future sea levels are very sensitive to changes in global temperatures resulting from uncertain greenhouse gases emissions scenarios [40]).
- RLPs and surrounding areas, based on data from FEMA’s Repetitive Loss Program. Areas surrounding RLPs (within 1,000m) were included in this index for three main reasons; first, RLPs are point occurrences, which have no area associated with them. By adding a buffer we can ensure that the area of the flooded parcel is include in the criterion; second, the accuracy of geographic coordinates of the RLPs data provided by FEMA is roughly 100m by 100m (for latitude and longitude); third, the exposure of areas adjacent to RLPs is also high and not all properties in the surrounding area may be insured, or have filed multiple claims against the NFIP, and therefore would be absent from the RLP dataset.

An overall FEI was calculated by summing up the values of individual flood exposure indicators within each grid cell according to the following equation:

FEI=F100+F500+RLP+CZ+SLR1+SLR2 (1)

where *F100* represents the 100-year floodplain score, *F500* represents the 500-year floodplain score, *WL* is the wetland score, *RLP* is the proximity to RLPs score, *CZ* is the coastal zone score, SLR1 is the area inside the MHHWM at the year 2100 score, and SLR2 is the areas projected to be inside the 100-year floodplain at the year 2100 score. FEI score values range from 0 to 6 (Table 1) and were scaled to range between 0 and 10 to balance its weight with that of the CPI, which also ranges from 0 to 10 (details below). The FEI score values were scaled according to the following feature normalization equation:

$$Flood\;Exposure\;Index=\frac{\left(X-Min\right)}{\left(Max-Min\right)}\;*\;10$$ (2)

where X is the value of the FEI for each grid cell before the normalization, Min is the minimum value of the index before normalization (i.e. 0), and Max is the maximum value of the index before normalization (i.e. 6).

**Table 1:** Flood Exposure Index (FEI).

FEI Components–data sources in parenthesis	Score
Area located within the 100-year Floodplain (FEMA)	1
Area located within the 500-year floodplain (FEMA)	1
RLPs and surrounding areas (1,000m buffer) (FEMA)	1
Area located in the California Coastal Zone (NOAA)	1
Area inside the projected MHHWM at the year 2100 (California Climate Change Center)	1
Area located inside the projected 100 year floodplain at the year 2100 (California Climate Change Center)	1
**Maximum possible FEI score**	**6 Points**

### 2. Conservation Priority Index (CPI)

There are many conservation prioritization schemes, developed by diverse institutions for multiple purposes [47–51]. Recognizing this variety, we demonstrate the flexibility of our approach by evaluating conservation priority in two ways. First, we used generally available raw spatial data representing natural resources and land cover to develop a unique CPI (Table 2). Second, we used TNC’s Priority Areas [47,52] as an alternative, pre-existing prioritization scheme[47].

**Table 2:** Conservation Priority Index (CPI).

CPI Components–data sources in parenthesis	Score
Area Located Inside Estuaries (U.S Fish and Wildlife Service)	1
Area Located Inside Wetlands (NOAA)	1
Salmon–Presence of Coho (WSC)	1
Salmon–Presence of Steelhead in the winter (WSC)	1
Salmon–Presence of Steelhead in the summer (WSC)	1
Salmon–Presence of Chinook in the fall (WSC)	1
Salmon–Presence of Chinook in the spring/summer (WSC)	1
Salmon–Presence of Chinook in the winter (WSC)	1
Area covered by Sand Dunes (TNC)	1
Areas with Urbanization Levels lower than 50% (NOAA)	1
**Maximum Possible CPI Score**	**10 Points**

The CPI was calculated based on the following components:

- Areas located inside estuaries (excluding water bodies), based on spatial data from the National Wetlands Inventory (NWI) produced by the U.S Fish and Wildlife Service [53].
- Areas Located inside wetlands (excluding deep water marine and lake interior), based on data from the Coastal Change Analysis Program (CCAP) produced by NOAA [54].
- Presence of salmonids, based on current and historical observations and expert opinion data from the Wild Salmon Center (WSC) [55]. There are three species of salmonids included in the study (Coho, Steelhead, and Chinook), which may be observed at different locations at different seasons of the year.
- Area covered by sand dunes, based on TNC’s northern California Current Ecoregion Assessment [56].
- Urbanization level, based on data from the Coastal Change Analysis Program (CCAP) produced by NOAA [54]. Areas with urbanization levels lower than 50% received a score of 1.

Every grid cell received a unique value for each Conservation Priority component. An overall CPI score was calculated by summing up conservation components scores according to the following equation:

CPI=ES+WL+SM1+SM2+SM3+SM4+SM5+SM6+S+U (3)

where *ES* represents the Estuaries score, *WL* is the Wetlands scores, *SM1* through *SM6* are the individual Salmonid scores, *S* is the Sand Dunes score and *U* is the Urbanization score. CPI score values can range from 0 to 10 (Table 2).

We have assigned uniform weights to each conservation criterion of the study. However, scores and weights can be adjusted to reflect conservation values of specific communities or conservation programs. A large proportion of CPI weight (60%) is based on the presence of three salmon species listed as either Endangered or Threatened. This specific prioritization scheme reflects the high restoration value of salmonid habitats and their special status under the Endangered Species Act (ESA). ESA mandates the identification and protection of all lands water and air necessary to recover endangered species [57]. Other applications of the method introduced here may consider other relevant criteria to calculate indices that reflect specific stakeholder’s interests.

In addition, to further illustrate the flexibility of our approach, we included an example of an existing conservation prioritization scheme in the study. We used TNC’s Priority Areas, [47] which were developed through comprehensive eco-regional assessments of species and habitat types [58]. Grid cells within a TNC Priority Area were given a score of 10. Because we use this index only to evaluate the spatial overlap between TNC Priority Areas and the FEI, our use of a Boolean data type is justified.

### 3. Spatial Model Description

All the spatial data layers described above were converted into raster format (grid cells), throughout the study area, each raster cell was attributed with three final scores representing Flood Exposure Index (Table 1), Conservation Priority Index (Table 2), and TNC’s Priority Areas.

The spatial extent of overlap between the FEI and the CPI was calculated by multiplying the CPI raster by the FEI raster, and can potentially vary from 0 to 100. Grid cells with scores equal to zero (indicating total absence of Conservation Priority, Flood Exposure, or both), resulted in a zero value, indicating no overlap between the two scores. The spatial extent of overlap between TNC’s Priority Areas and the FEI was calculated in a similar way, by multiplying TNC’s Priority Area raster values (0 or 10) by the FEI (0 to 10) in each grid cell. The final step in the calculation was the multiplication of the number of grid cells in the resulting raster by the area of each grid cell.

Additionally, we analyzed the distribution of Repetitive Loss Properties throughout our study areas and how their distribution relates to our three categories (Conservation Priority, Flood Exposure and TNC’s Priority Areas). Finally, we chose Sonoma County, the county with the highest number of repetitive loss claims in California, to conduct a focused case study. The same methodology used in the broader study area was applied in the case study.

## Results

We found that there are at least 11,243 km^2^ in coastal California which represent both flood exposure reduction and conservation value, and where property/structure buyouts and habitat restoration projects would meet multiple objectives. This area covers almost 12% of the total study area of 94,500km^2^, with the extent of land decreasing as each score increases.

We scored areas in the 21 coastal counties of California based on flood exposure and conservation priority, and applied the spatial model described above to calculate the areal extent of overlap between them in order to prioritize potential areas for multiple-benefit projects, which accomplish both flood mitigation and conservation or restoration of natural habitats.

**Fig 1:**
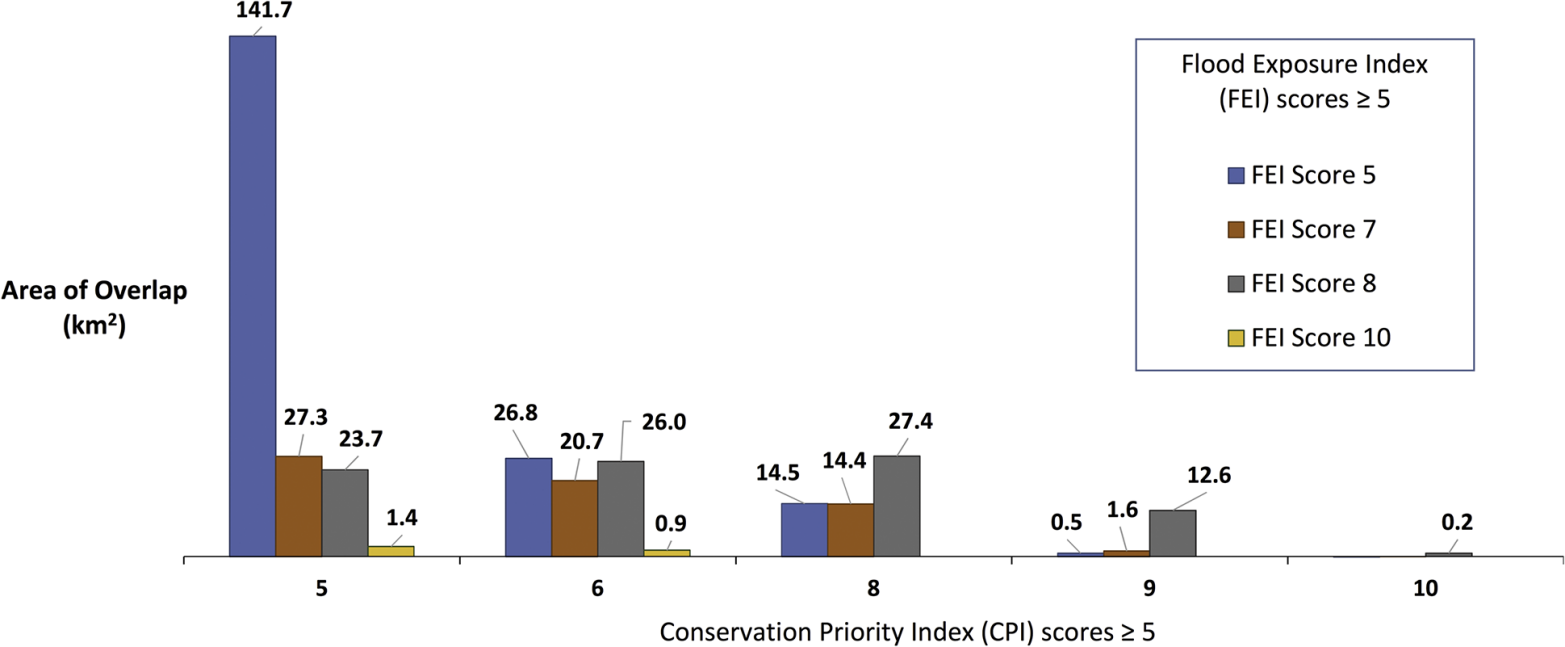
Area of overlap between CPI and FEI scores greater than or equal to 5. CPI scores equal to 7 and FEI scores equal to 6 and 9 did not occur in the study area, therefore are not shown in Fig 1.

Areas that scored at least 1 point for both Flood Exposure and Conservation Priority intersect extensively in the 21 coastal counties of California. For example, the area of overlap between the Flood Exposure Index greater than 1 and the Conservation Priority Index equal to or greater than 5, is 954 km^2^. The highest priority areas for both indices (FEI ≥5 and CPI ≥ 5) covers 340 km^2^ (total area calculated from Fig 1).

**Fig 2:**
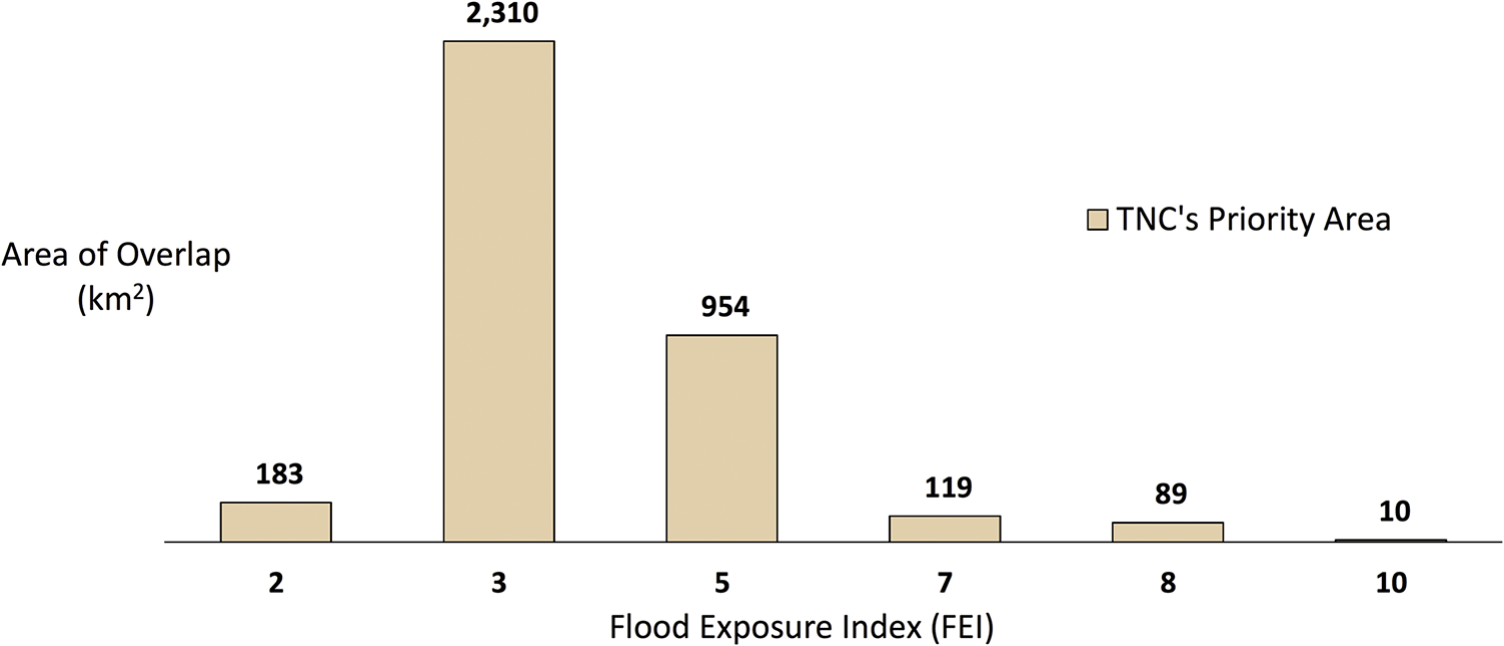
Area of overlap between TNC's Priority Areas and FEI greater than or equal to 1. FEI scores equal to 1, 4, 6 or 9 did not occur in the study area, therefore are not shown in Fig2.

Additionally, we substituted a score based upon a pre-existing conservation prioritization scheme (TNC’s Priority Areas) for the CPI briefly described above (see Materials and Methods). In coastal California, the overlap between TNC’s Priority Areas and areas where the FEI scores greater than 0 covers an area of 3,665km^2^ (total area calculated from Fig 2). This extent is much smaller than the total coverage of TNC’s Priority Areas (more than 40,000 km^2^), across the entire study region. If we focus only on areas where TNC’s Priority Areas overlap with high FEI scores (overlap scores > 50), the resulting area is roughly 218 km^2^, spread across 15 coastal counties. Moreover, if we focus on areas where TNC’s Priority Areas overlap with very high Flood Exposure score (overlap score equal 100) the resulting area is only about 10 km^2^, spread across 8 coastal counties.

The area of intersection between CPI and FEI (11,243km2) is larger than the area of intersection between TNC’s Priority Areas and the FEI (3,664km2). Broadly, however, this indicates that the model can be applied to reflect distinct conservation priorities of the user.

**Fig 3:**
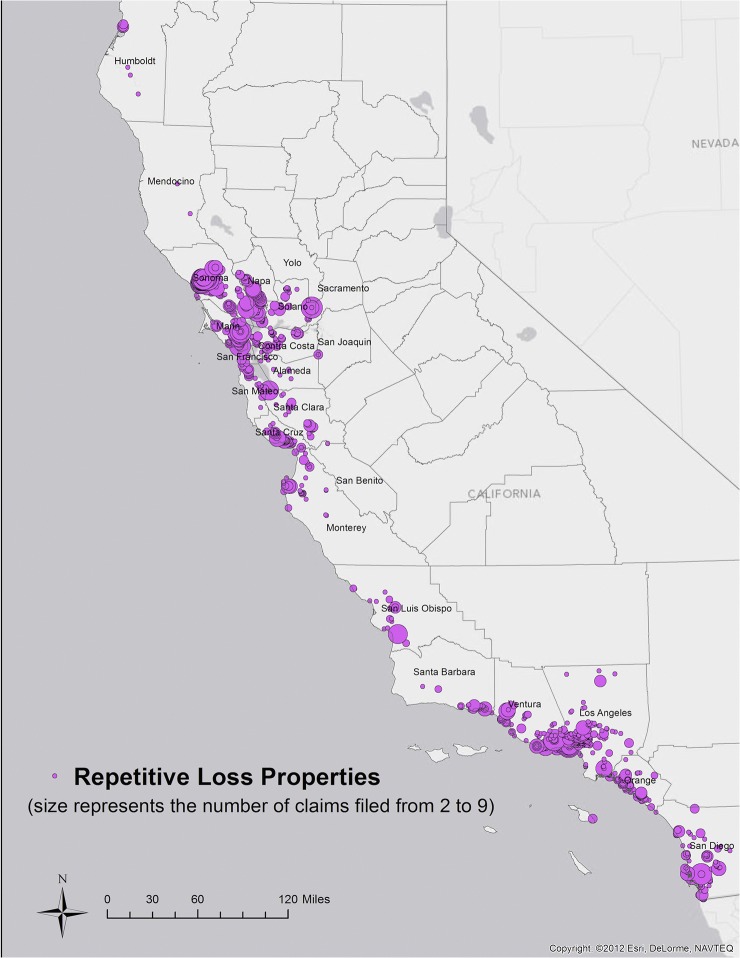
Repetitive Loss Properties throughout California (fuchsia circles). The size of each circle increases with number of losses (ranging from 2 to 9 losses) [59].

There are over 3,200 Repetitive Loss Properties located throughout California (Fig 3). In the 21 coastal counties included in the study area, 2,395 Repetitive Loss Property owners filed 6,794 claims against the NFIP from 1978 to April 2010. This represents 77% of the total number of Repetitive Loss Properties and 79% of the total number of claims in the state for the same period.

Sixty-six percent of the Repetitive Loss Properties (1,589 properties) located in our study area, are situated in areas with a CPI score greater than 0. Roughly 18% of all Repetitive Loss Properties (440 properties) are located in areas with a CPI score of at least 5. Approximately 44% of all Repetitive Loss Properties in the state (1,051 properties) co-occur with TNC’s Priority Areas.

### Sonoma County Case Study

**Fig 4:**
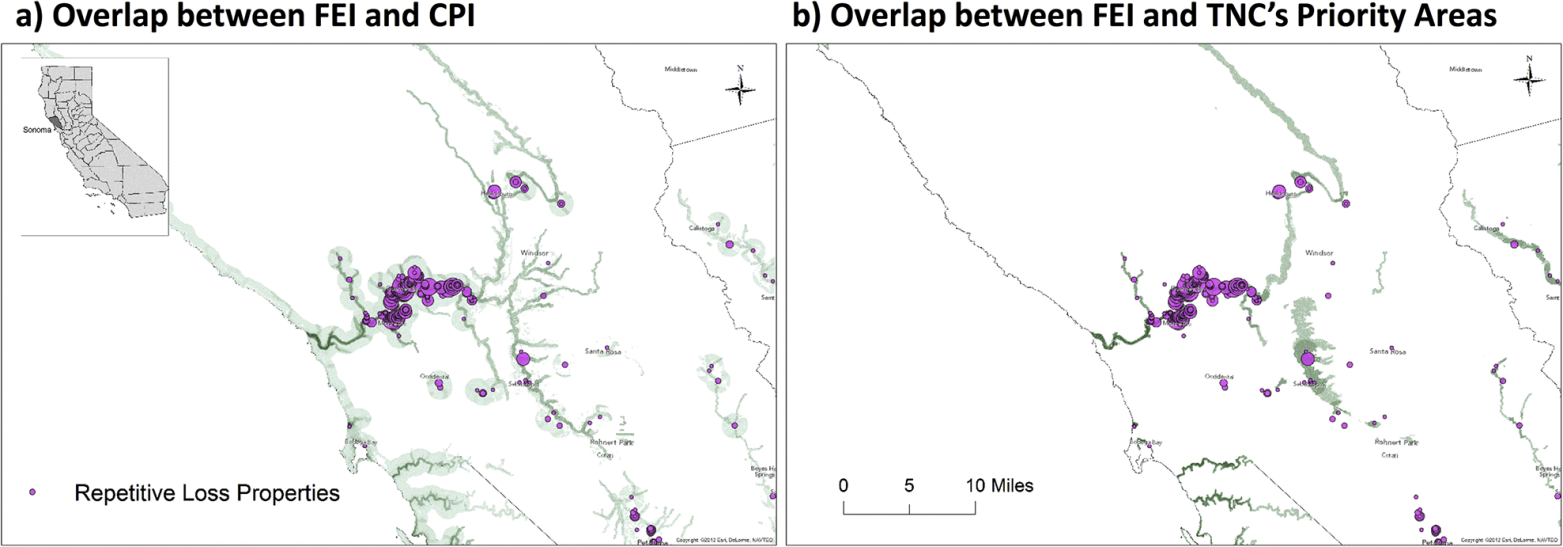
RLPs in Sonoma County and a) overlap between the FEI and CPI; b) overlap between the FEI and TNC’s Priority Areas. Darker green indicates areas where higher indices values overlap. Fuchsia circles represent the locations of RLPs (as of April 2010). The size of the circles represent the total number of flood claims filed (from 2 to 9) by each RLP.

Here we highlight results from Sonoma County, which is a hotspot of repetitive loss in California (Table 3). Flood Exposure and Conservation Priority intersect to a very significant degree in Sonoma County. The area of intersection between FEI scores greater than 1 and CPI scores greater than 1, is 564 km^2^—roughly 12.6% of the county area (Fig 4, left panel). The intersection of areas having a FEI score greater than 0 and TNC’s priority areas in Sonoma covers an area of 128 km^2^, roughly 3% of the county area (Fig 4, right panel). The overlap between TNC’s Priority Areas and FEI score greater than or equal to 5 is about 42km^2^.

**Table 3:** Distribution of Repetitive Loss properties and Claims in California by County.

County	Number of RLPs	% of RLPs	Number of Claims	% of Claims
**Sonoma**	**853**	**36%**	**2734**	**40%**
Los Angeles	434	18%	1147	17%
Marin	192	8%	532	8%
Napa	114	5%	346	5%
Monterey	112	5%	248	4%
San Diego	106	4%	282	4%
Orange	101	4%	251	4%
Santa Cruz	89	4%	254	4%
Ventura	77	3%	201	3%
Santa Barbara	74	3%	165	2%
Contra Costa	66	3%	169	2%
Solano	48	2%	125	2%
San Mateo	32	1%	83	1%
San Luis Obispo	32	1%	87	1%
Santa Clara	29	1%	84	1%
Alameda	15	1%	32	0%
Other	36	2%	86	1%
**Total**	**2,395**	**100%**	**6,794**	**100%**

Table 3 only contains data for the 21 coastal counties in California, from January 1978 to April of 2010 (Source: FEMA).

Nearly all of the Repetitive Loss Properties in Sonoma (95.3% or 813 properties) are located in areas where Flood Exposure and Conservation Priority indices overlap. The total number of properties where there is an overlap between FEI and TNC’s Priority Areas is slightly lower but still large (83.1% or 709 properties). The highest priority areas in Sonoma County may be located where the highest FEI (10) overlap with CPI greater than or equal to 5. In Sonoma County, 32 Repetitive Loss Properties are in such areas and have filed at least 95 combined claims.

## Discussion

Our results demonstrate that there can be significant synergies between the objectives of flood exposure reduction and those of habitat conservation and restoration projects. We have identified and prioritized high-leverage sites for such multi-objective projects in California, and our approach can be applied to any geography where floods have repeatedly resulted in financial and human losses, and where critical natural resources are present. For the purposes of this discussion, a multi-objective project should be understood as one in which structures associated with a RLP are removed from the floodplain to permit habitat restoration.

Buyouts of property and structures have been a part of the FEMA’s overall risk reduction strategy since the 1980s [60], but a number of factors–including an understandable hesitation to abandon homes and neighborhoods–have limited its application [61]. Removal of structures from floodplains has ecological benefits in addition to hazard mitigation benefits, including increases in wetland acreage, restoration of wildlife habitat and reconnection of fragmented habitat [62]. An important added benefit of wetlands restoration is that, in some cases, restored floodplains may also function as natural flood mitigation infrastructure [21,63].

Communities are increasingly considering the application of buyouts as a strategy to reduce their long term risks and therefore need to prioritize parcels for the application of limited funds [64]. Following super storm Sandy in October 2012, New York State conducted a needs assessment to prioritize the allocation of federal disaster recovery funds, and 34% of responders (totaling 2,582 people) indicated interest in a buyout of their home [64]. The present study demonstrates a prioritization scheme that would support the elimination of flood exposure for the target parcel (and possibly to neighboring parcels as well), restoration of natural resources, and efficient use of limited governmental funds.

Our analysis of Sonoma County, California’s epicenter of repetitive losses with 36% of all Repetitive Loss Properties (853) and 40% of all individual claims (2,734) filed in the study area (as of April 2010), is particularly illustrative of this principle. As of June of 2010, Sonoma County had received more than $53 million in payments from grants intended to mitigate flooding on Repetitive Loss Properties; this value represents more than 30% of the total amount of RLP grants received by California for the same period ($171.7 million) [36]. Meanwhile, Sonoma County has critical biodiversity conservation objectives, including restoration of steelhead trout, Chinook and Coho salmon, all of which are listed as threatened or endangered under the federal Endangered Species Act [38]. Our results suggest that Sonoma County’s efforts to restore salmonid habitat and its efforts to reduce Flood Exposure are very well aligned.

There is a significant need for conservation and restoration of coastal habitats, but limited resources available for accomplishing these goals. Specifically, the National Oceanic and Atmospheric Administration’s Restoration Center has a planned budget of $42 million for 2015, [23]. By contrast, FEMA’s obligated funds for the Hazard Mitigation Grant Programs in 2013, exceeded $700 million [24].Nonprofit organizations, federal, state, and local agencies, and other decision makers should use analyses like the one presented here to strengthen the case for the application of hazard mitigation funds to acquire properties or engage in restoration in areas with both high flood exposure and high conservation value.

The multiple benefits to this approach include: elimination of risk for the target parcel, reduction of the financial impact to NFIP of repeated flood claims, and restoration of land to a more natural condition.

The qualitative approach proposed here could be further enhanced by additional case studies that should focus on Los Angeles, Marin, Napa and Monterey Counties, which combined account for 36% of RLPs and 33% of the total number of claims in the study area. Additionally, including a third index scoring socioeconomic vulnerability would provide valuable insight to the potential benefits or consequences of buyout projects to disadvantaged demographics. Finally, our proposed approach should be applied to other coastal states of the country, utilizing substitute relevant local conservation criteria (e.g. sea grass and mangroves instead of salmonids could be used in Florida).

## Supporting Information

S1 Appendix Detailed Description of Repetitive Loss Properties and Recent NFIP Regulatory Updates. (DOCX)
