# Supplementary material for: Aligning Natural Resource Conservation and Flood Hazard Mitigation in California
Source: PLoS One. 2015 Jul 22;10(7):e0132651. doi: 10.1371/journal.pone.0132651 (PMC4511615; doi:10.1371/journal.pone.0132651)
Supplement: S1 Appendix — (DOCX) [file pone.0132651.s001.docx]

**Appendix S1.** Detailed Description of Repetitive Loss Properties and Recent Regulatory Updates.

**Federal Emergency Management Agency (FEMA)’s Definition of Repetitive Loss Properties; the Biggert Waters Flood Insurance Reform Act of 2012 (BW-12); and the Homeowner Flood Insurance Affordability Act of 2014.**

The database used in this study contains the locations of Repetitive Loss Properties based on their former definition. Prior to July of 2012, Repetitive Loss Properties were classified as any federally insured property for which two or more claims, of more than $1,000 each, were paid by the NFIP within any rolling ten-year period (1) .As of July of 2012, through the approval of the Biggert-Waters act, FEMA has redefined RLPs as structures covered by flood insurance under the NFIP that: (a) incurred flood-related damage on 2 occasions, in which the cost of the repair, on the average, equaled or exceeded 25 percent of the market value of the structure at the time of each flood; and (b), at the time of the second incidence of flood-related damage, the contract for flood insurance contains increased cost of compliance coverage (2).

In an effort to bring stability and fiscal soundness to the national flood insurance program, in July of 2012, Congress approved the Biggert Waters Flood Insurance Reform Act (BW-12). Among other provisions, BW-12 reauthorized NFIP for an additional 5 years (from 2013 to 2017) and defined a gradual adjustment of insurance rates to reflect true risk. BW-12 also consolidated two former Hazard Mitigation Grant Programs - the Repetitive Flood Claims (RFC) and the Severe Repetitive Loss (SRL) grant programs - under the Flood Mitigation Assistance (FMA) grant program. Reacting to strong public reaction and concerns that the new flood insurance rates triggered by BW-12 would affect the housing market, as well as drive home owners from their properties, in March of 2014, congress enacted the Homeowner Flood Insurance Affordability Act of 2014. The 2014 Act modified and repealed several provisions of BW-12 by implementing measures including: limiting the increase of annual flood insurance premiums to 18%, repealing any rate increases triggered by property sales or the acquisition of new and voluntary flood insurance policies, refunding select policy holders for recent rate increases, and authorizing additional funds for the National Academy of Sciences to complete an affordability study (2).

1. FEMA, Mitigation H, Program G, Program PM (2013) *Mitigation Assistance Unified Guidance* Available at: http://www.fema.gov/media-library-data/1381842520166-4d0b88314cfaa2b7e114391ce6ff2d73/508_FINAL_Guidance_09112013.pdf.

2. Flood Insurance Reform | FEMA.gov Available at: http://www.fema.gov/flood-insurance-reform [Accessed April 30, 2014].
